# Circulating tumor cells, tumor-derived extracellular vesicles and plasma cytokeratins in castration-resistant prostate cancer patients

**DOI:** 10.18632/oncotarget.25019

**Published:** 2018-04-10

**Authors:** Afroditi Nanou, Frank A.W. Coumans, Guus van Dalum, Leonie L. Zeune, David Dolling, Wendy Onstenk, Mateus Crespo, Mariane Sousa Fontes, Pasquale Rescigno, Gemma Fowler, Penny Flohr, Christoph Brune, Stefan Sleijfer, Johann S. de Bono, Leon W.M.M. Terstappen

**Affiliations:** ^1^ Department of Medical Cell BioPhysics, MIRA Institute, University of Twente, Enschede, the Netherlands; ^2^ Department of Biomedical Engineering and Physics, Academic Medical Center, University of Amsterdam, the Netherlands; ^3^ Department of General, Visceral and Pediatric Surgery, University Hospital and Medical Faculty of the Heinrich-Heine University, Düsseldorf, Germany; ^4^ Department of Applied Mathematics, MIRA Institute and Faculty of EEMCS, University of Twente, Enschede, the Netherlands; ^5^ Department of Medical Oncology, Erasmus MC – Cancer Institute, Rotterdam, The Netherlands; ^6^ Division of Clinical Studies, The Institute of Cancer Research, London, United Kingdom; ^7^ Prostate Cancer Targeted Therapies Group, The Royal Marsden NHS Foundation Trust, London, United Kingdom

**Keywords:** circulating tumor cells (CTCs), tumor-derived Extracellular Vesicles (tdEVs), cytokeratin 18 (CK18), caspase-cleaved cytokeratin 18 (ccCK18), castration-resistant prostate cancer (CRPC)

## Abstract

**Purpose:**

The presence of Circulating Tumor Cells (CTCs) in Castration-Resistant Prostate Cancer (CRPC) patients is associated with poor prognosis. In this study, we evaluated the association of clinical outcome in 129 CRPC patients with CTCs, tumor-derived Extracellular Vesicles (tdEVs) and plasma levels of total (CK18) and caspase-cleaved cytokeratin 18 (ccCK18).

**Experimental Design:**

CTCs and tdEVs were isolated with the CellSearch system and automatically enumerated. Cut-off values dichotomizing patients into favorable and unfavorable groups of overall survival were set on a retrospective data set of 84 patients and validated on a prospective data set of 45 patients. Plasma levels of CK18 and ccCK18 were assessed by ELISAs.

**Results:**

CTCs, tdEVs and both cytokeratin plasma levels were significantly increased in CRPC patients compared to healthy donors (HDs). All biomarkers except for ccCK18 were prognostic showing a decreased median overall survival for the unfavorable groups of 9.2 vs 21.1, 8.1 vs 23.0 and 10.0 vs 21.5 months respectively. In multivariable Cox regression analysis, tdEVs remained significant.

**Conclusions:**

Automated CTC and tdEV enumeration allows fast and reliable scoring eliminating inter- and intra- operator variability. tdEVs provide similar prognostic information to CTC counts.

## INTRODUCTION

The presence of Circulating Tumor Cells (CTCs) in Castration-Resistant Prostate Cancer (CRPC) as detected by the CellSearch system is associated with poor outcome compared to patients without detectable CTCs [1–6]. Previously, we showed that the presence of small and large tumor microparticles with or without nucleus, positive for Epithelial Cell Adhesion Molecule (EpCAM) and Cytokeratin (CK) and negative for the leukocyte marker CD45 are also associated with poor outcome in CRPC patients [7]. These tumor microparticles can also be measured using the CellSearch system but do not meet the stringent criteria for CTCs. In the present study, we investigate the clinical relevance of both EpCAM+ CK+ CD45- tumor microparticles without a nucleus in blood, defined here as tumor-derived Extracellular Vesicles (tdEVs) and soluble cytokeratins in plasma of CRCP patients. Our interest in cytokeratin plasma levels arises from the fact that one of the prerequisites for a cell and an EV isolated by the CellSearch system in blood to be defined as CTC and tdEV respectively is their cytokeratin expression because of their epithelial origin. Therefore, we additionally investigated whether soluble cytokeratins present in plasma may be associated with clinical outcome and applied as a surrogate biomarker. Measurements of plasma levels of cytokeratin 18 (CK18) and caspase-cleaved cytokeratin 18 (ccCK18) were performed using M65 and M30 ELISAs respectively [8]. CTCs and tdEVs were imaged with the CellSearch system [9] and automatically enumerated with ACCEPT software to avoid interoperator variability [10]. Association of CTCs, tdEVs, CK18 and ccCK18 with clinical outcome in advanced CRPC patients was assessed by Kaplan–Meier plots of Overall Survival (OS), uni-, and multi- variable Cox regression analyses.In this study, two data sets were used: a retrospective data set of 84 patients to determine the cut-off values of CTCs and tdEVs for favorable and unfavorable prognosis and a prospective data set of 45 patients to validate the selected cut-off values. The plasma samples of the two data sets were collected in a different way; hence, different cut-off values for CK18 and ccCK18 were used for each one of them.

## RESULTS

### Patient characteristics

Blood draws of the CRPC patients consisting the retrospective data set (IMMC 38 study) were performed between October 2004 and February 2006. Their average age was 70 years (range 49–87 years). Blood draws of the CRPC patients consisting the prospective data set were performed between March 2015 and August 2015. Their average age was 68 years (range: 49–83 years). The values of serum markers (PSA, LDH, ALP,Alb,Hb), age, Gleason score and ECOG performance status for all patients of both data sets are shown in Supplementary Table 1. The site of metastases, prior radiation, surgery and treatment of both data sets are summarized in Supplementary Table 2. The prospective data set seem to have more advanced disease compared to the retrospective data set, since significantly more patients underwent chemotherapy (91.1% versus 35.7%). Furthermore, 82.2% and 46.7% of the patients of the prospective data set were already resistant to abiraterone and enzalutamide respectively, indicating more progressed disease compared to the retrospective dataset, where some patients were still undergoing hormone therapy.

### Number of CTCs and tdEVs in 7.5 mL of blood

**Figure 1:**
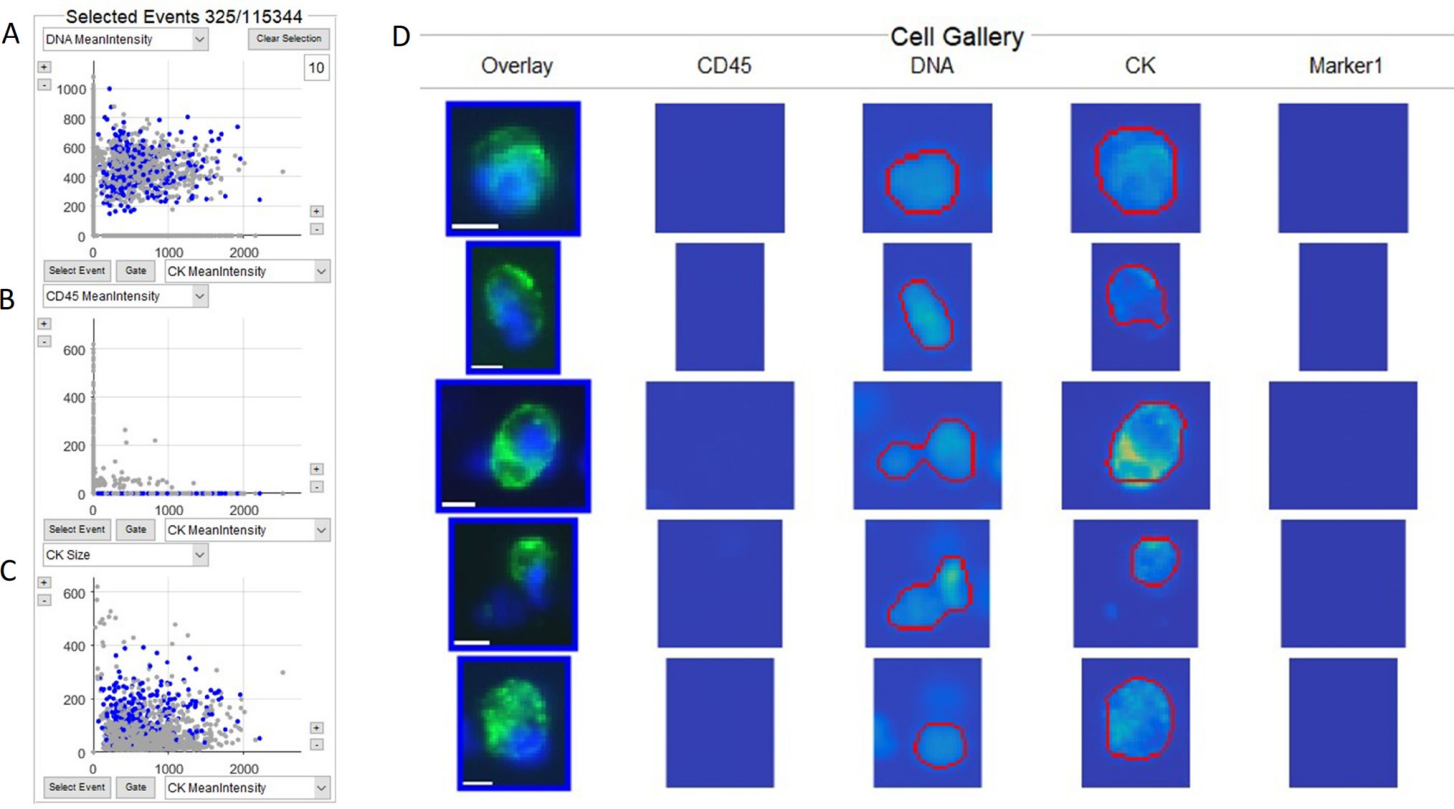
ACCEPT display of CTCs identified in a CRPC patient Three scatterplots (**A**–**C**), where the objects that fall within the definition of the CTC gate (Mean Intensity CD45 ≤ 5, Mean Intensity DNA > 45, Mean Intensity CK > 60, Mean Intensity Marker 1 ≤ 5, Mean Intensity Marker 2 ≤ 5, 16 ≤ Size CK ≤ 400, DNA overlay CK > 0.2), are shown as blue dots and those that fall outside of the gate are shown as grey dots. The total number of objects (115,344) identified and the number of objects within the gate (325) are shown on top of Panel A. (**D**) shows the thumbnail images of five objects that fall within the gate. The red lines in the thumbnail identifies the area in the image in which ACCEPT identifies contours of the object in each channel. Thumbnails that do not show red contours indicate that no object in this channel could be detected. In the overlay thumbnails, DNA is represented in blue, cytokeratin (CK) in green and CD45 in red. As no CD45 is detected in these images, no red is shown in the overlay. Scale bar indicates 6.4 μm.

CTCs (Figure 1) and tdEVs (Supplementary Figure 1) were enumerated from the stored digital images using ACCEPT software. We compared manually defined CTCs by a human operator using standard CellSearch criteria for recognition (CK+, CD45-, DAPI+, >4 μm) versus the automated CTCs defined by a preconfigured quantitative ACCEPT gate for 129 CRPC patients and 16 healthy controls, see Supplementary Figure 2. Linear regression resulted in “automated CTCs” = 0.82 “manual CTCs” + 0.49 (R^2^ = 0.882).

**Figure 2:**
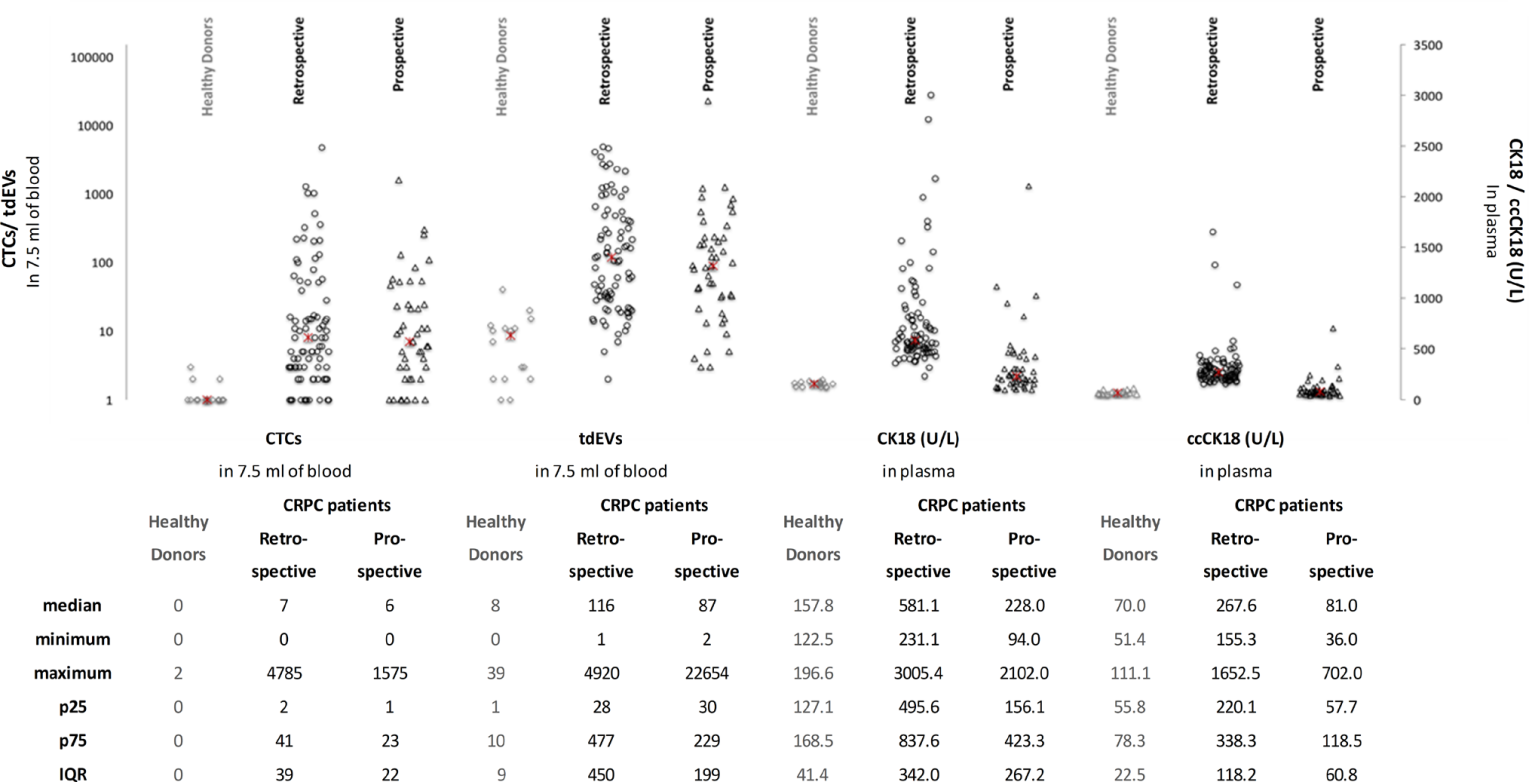
Number of automated CTCs and tdEVs in 7.5 mL of blood and plasma levels of CK18 and ccCK18 in 16 HDs, a retrospective data set of 84 CRPC patients and a prospective data set of 45 CRPC patients The median, minimum, maximum, 25 and 75 percentiles (p25, p75 respectively) and interquartile range (IQR) values of CTCs, tdEVs, CK18 and ccCK18 are shown below the scatter plot. The median value of each variable is indicated as red × in the scatter plot.

The median, min, max, 25 and 75 percentiles (p25, p75 respectively) and InterQuartile Range (IQR) values of CTCs and tdEVs detected in the 16 healthy controls, the retrospective data set of 84 and the prospective data set of 45 CRPC patients are depicted in Figure 2. The number of CTCs and tdEVs were significantly higher in the CRPC patients as compared to HDs (both *p <* 0.001, Mann-Whitney *U* test). There were no significant differences for CTCs nor for tdEVs between the two data sets (*p =* 0.81 for CTCs and *p =* 0.32 for tdEVs, Mann Whitney *U* test).

Spearman’s rho correlation test showed that CTCs and tdEVs of the full data set of 129 CRPC patients were correlated (Spearman’s R = 0.856, *p <* 0.01). The scatterplot of that data is shown in Supplementary Figure 2.

### CK18 and ccCK18 concentrations in plasma samples

The determined CK18 and ccCK18 concentrations of 16 HDs, 84 retrospective and 45 prospective CRPC patients are illustrated in Figure 2. The concentrations of CK18 and ccCK18 were significantly higher in the CRPC patients compared to the healthy controls (*p <* 0.001, Mann-Whitney *U* test). Unexpectedly, the concentrations of CK18 and ccCK18 were also significantly higher in retrospective data set as compared to the prospective one (*p <* 0.001, Mann-Whitney *U* test). Further investigation (Supplementary Figure 4) revealed that the main contributor was the use of EDTA plasma in IMMC38 study versus the CellSave plasma samples in the prospective study.

Given the different values obtained in the two data sets, correlations between CTCs, tdEVs, CK18, ccCK18 and the serum markers of the patients were evaluated within each data set and not in the full data set, Supplementary Table 3. Using Spearman’s Rho test, CTCs and tdEVs were strongly correlated.

### Determination of cut-off values for CTCs, tdEVs, CK18 and ccCK18

ROC curves were generated on the retrospective data set to determine the cut-off values for CTCs, tdEVs, CK18, and ccCK18 dichotomization into favorable and unfavorable patient groups for graphical representation. We set dichotomization cut-off values for CTCs, tdEVs, CK18, and ccCK18 on the value that led to equal sensitivity and specificity (value of biomarker for which absolute (sensitivity-specificity) was minimum), Supplementary Figure 3. The cut-off values were 5 for CTCs, 105 for tdEVs, 576 U/L for CK18 and 265 U/L for ccCK18. tdEVs performed the best in terms of sensitivity and specificity to predict OS of CRPC patients (having the largest AUC and the highest significance), followed by CTCs and CK18.

Because of the large differences of CK18 and ccCK18 concentrations between the two data sets, we also determined dichotomization cut-off values on the prospective data set, these were 232 U/L for CK18 (was 576 U/L) and 81 U/L for ccCK18 (was 265 U/L).

### CTCs, tdEVs, CK18, and ccCK18 versus overall survival of CRPC patients

**Figure 3:**
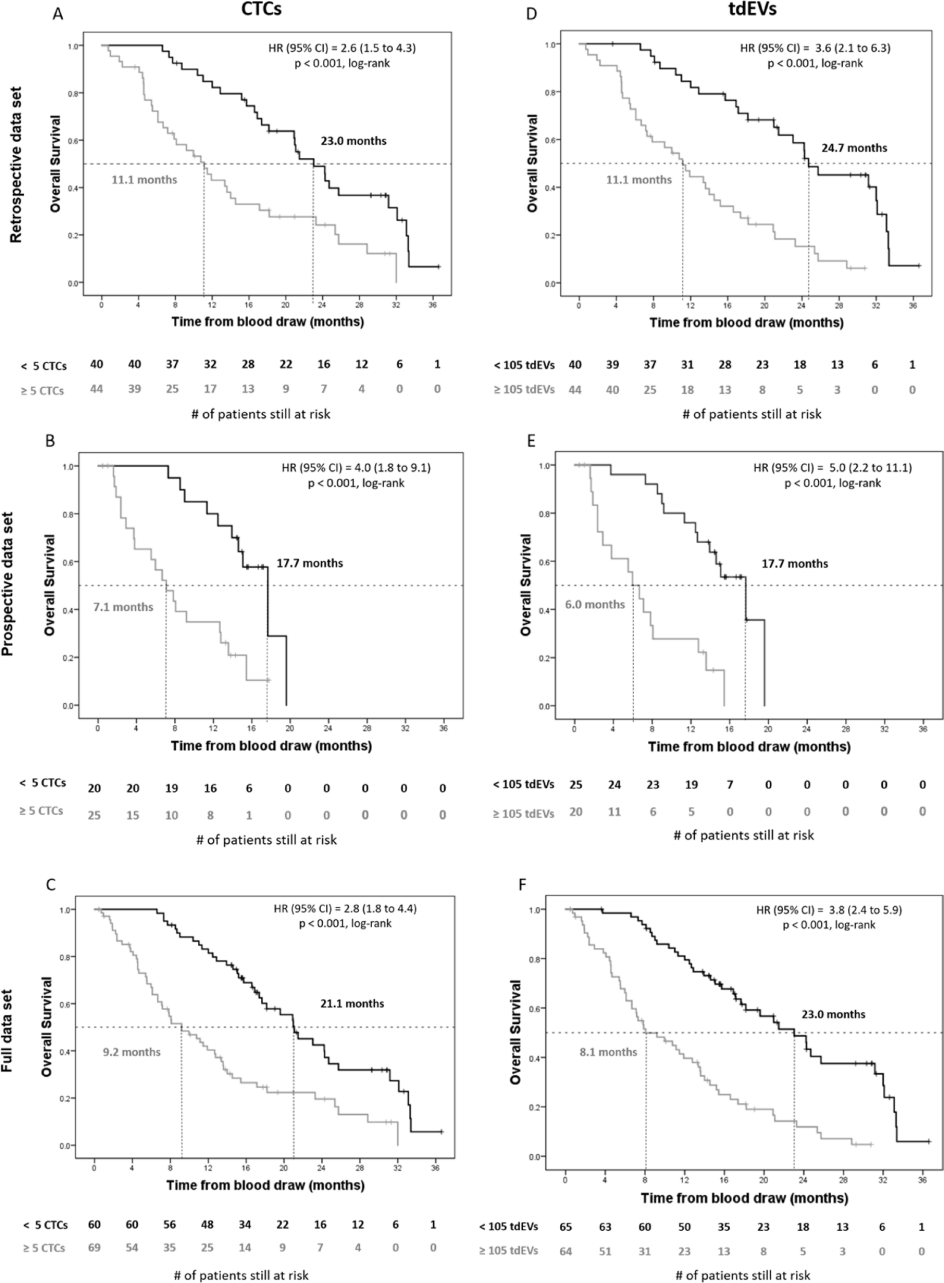
Kaplan–Meier plots of overall survival of retrospective, prospective and full data set of CRPC patients for automated CTCs and tdEVs Kaplan–Meier plots of overall survival of CRPC patients before initiation of therapy for automated CTCs (Panels **A**–**C**) and tdEVs (Panels **D**–**F**). The retrospective (*n* = 84), prospective (*n* = 45), and full (*n* = 129) data sets are shown in panels A/D, B/E, and C/F respectively. Patients were dichotomized into unfavorable (grey lines) and favorable groups (black lines) on a cut-off value of 5 for CTCs and 105 for tdEVs in 7.5 mL of blood. Vertical tick marks indicate censored patients. The number of patients at risk in each group is shown under the horizontal axis. The median overall survival for each group, the Cox hazard ratio (HR), and significance (log-rank p) are indicated in each panel.

Kaplan–Meier plots for patients with favorable and unfavorable CTC and tdEV counts are shown in Figure 3. Median OS of patients in the unfavorable CTC group was 9.2 months, versus 21.1 months in the favorable CTC group (*p <* 0.001). Similarly for tdEVs, median OS of patients in the unfavorable tdEV group was 8.1 versus 23.0 months in the favorable tdEV group (*p <* 0.001).

**Figure 4:**
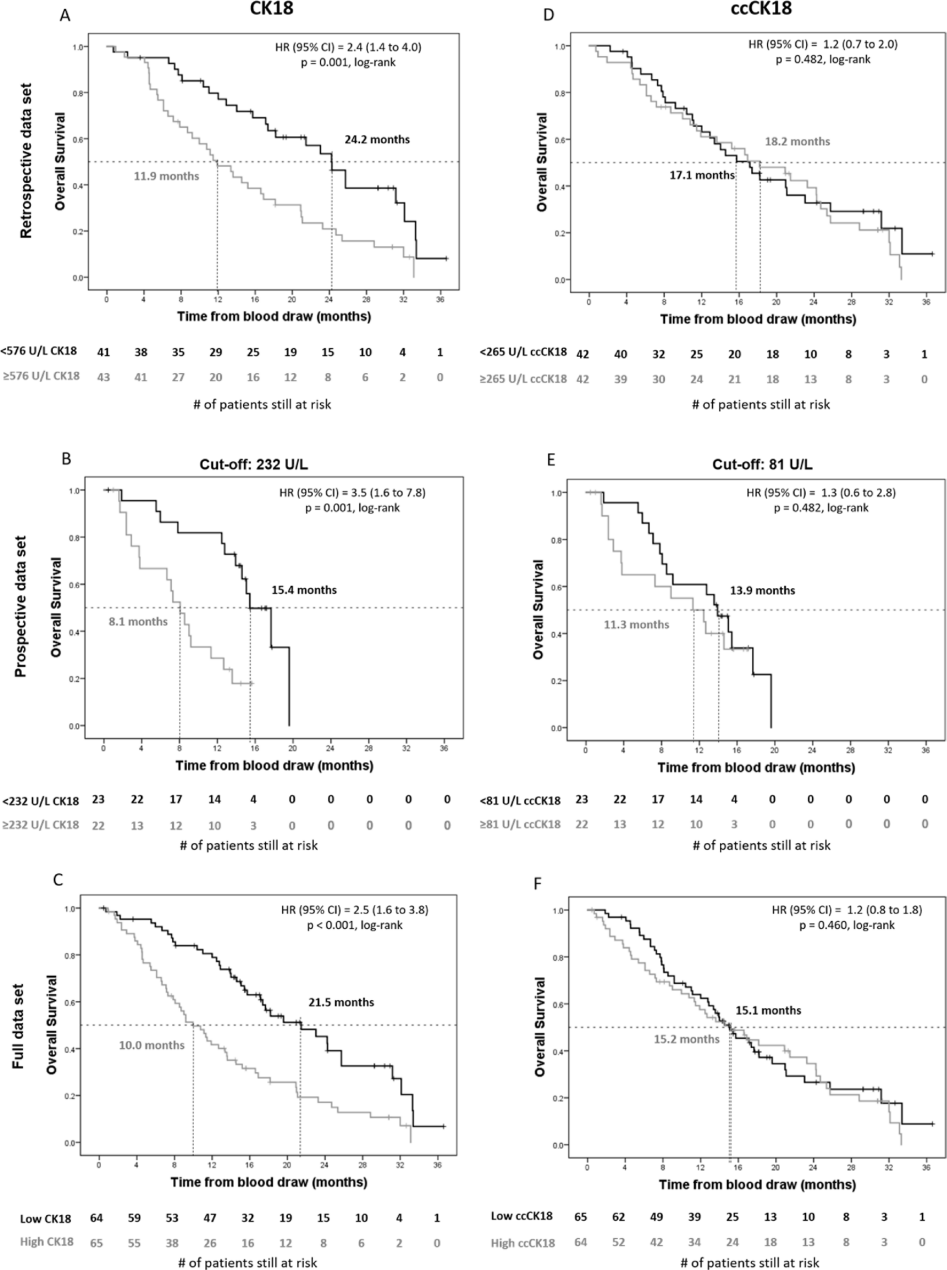
Kaplan–Meier plots of overall survival of retrospective, prospective and full data set of CRPC patients for plasma levels of CK18 and ccCK18 Kaplan–Meier plots of overall survival of CRPC patients before initiation of therapy for total plasma cytokeratin 18 (CK18, Panels **A**–**C**) and caspase-cleaved plasma cytokeratin 18 (ccCK18, Panels **D**–**F**) in retrospective (*n* = 84, Panels A, D), prospective (*n* = 45, Panels B, E) and full (*n* = 129, Panels C, F) data sets. Patients were dichotomized into unfavorable (grey lines) and favorable groups (black lines) on a cut-off value of 576 U/L CK18 and 265 U/L ccCK18 for panels A, D. Patients were dichotomized on a cut-off value of 232 U/L CK18 and 81 U/L ccCK18 for panels (B, E). Dichotomization on the full data set (Panels C, F) was done using the different cut-off values for each data set. Vertical tick marks indicate censored patients. The number of patients at risk in each group is shown under the horizontal axis. The median overall survival for each group, the Cox hazard ratio (HR), and significance (log-rank p) are indicated in each panel.

Kaplan–Meier plots for CK18 and ccCK18 are shown in Figure 4. The retrospective data set is shown in panels A/D. The prospective data set is shown in panels B/E, and the full data set is shown in panels C/F. The two data sets were dichotomized using different cut-off values, because the plasma samples were collected in a different way. However, in both data sets CK18 was prognostic of OS with the HRs as well as the relative number of patients in favorable and unfavorable groups very similar between the two data sets. For the retrospective data set, the median OS of patients with unfavorable CK18 was 11.9 months, compared to 24.2 months in the favorable group (*p =* 0.001) with a HR of 2.4. Similarly, for the prospective data set, the patients in the unfavorable CK18 group had significantly lower survival (8.1 versus 15.4 months in the favorable group, *p =* 0.001) with a HR of 3.5. For ccCK18 no significant difference (*p =* 0.48) in OS could be observed between the two groups regardless the cut-off value used. The summarized HRs of all variables are summarized in Supplementary Table 4.

### Multivariable Cox proportional hazards regression analyses

Table 1 shows the final multivariable Cox model selected which included variables CTCs, tdEVs, CK18, prostate specific antigen (PSA), alkaline phosphatase (ALP), lactate dehydrogenase (LDH), albumin (Alb), hemoglobin (Hb) and age. Of the known prognostic variables, LDH, albumin and hemoglobin were all selected in the final model. There was no evidence that either CK18 or ccCK18 were predictive of overall survival after LDH was included in the multivariable model. CTCs were highly correlated with tdEV (Pearson’s rho = 0.79 for the transformed variables) and were not included in the final multivariable model after tdEV was the first variable selected. At 24 months, Uno’s C-Index was significantly higher in the multivariable model with tdEV (C-Index = 0.77) compared to the multivariable model which included LDH, albumin and hemoglobin (C-Index = 0.73; Difference = 0.04; 95% CI = 0.01 to 0.06; *p =* 0.006) suggested that tdEV improved prognostic prediction. There was no evidence of a difference in the prognostic abilities of the model which included CTCs, LDH, albumin and hemoglobin (C-Index = 0.77) compared to the model which included tdEVs (Difference = –0.002; 95% CI: –0.02 to 0.01; *p =* 1.00)

**Table 1:** Multivariable cox proportional hazards regression analysis for full data set of CRPC patients

Variables in equation	HR	95%	CI	*p*-value
Albumin (g/dl)	0.38	0.21	0.67	0.001
Hemoglobin (g/l)	0.81	0.69	0.94	0.006
tdEV (ln(0.0001 + count/1000))	1.30	1.11	1.51	0.001
LDH (ln(0.001 + U/L/100))	1.84	1.09	3.12	0.023

(*N* = 118, 11 cases were dropped due to missing values).

## DISCUSSION

Rapid advances in drug development and treatment of cancer patients increase the necessity for new biomarkers to assess their prognosis and response to therapy accurately and in a timely fashion. In the management of CRPC patients, adequate response to therapy is challenging as the traditional Response Evaluation Criteria in Solid Tumors [11] frequently cannot be applied. The presence or absence of CTCs has emerged as a powerful biomarker to assess prognosis and therapy response [1–5]. Although the CTC numbers measured are extremely low, several ring studies have been conducted and demonstrated the robustness of the test, though a certain level of operator bias cannot be completely eliminated [12–15]. In the original studies conducted with the CellSearch system, patient groups were divided in those with less or more than 5 CTCs per 7.5 mL of blood and shown to have different clinical outcomes [1–5]. However, recently it was demonstrated that the actual number of CTCs -and not just a count above or below a selected cut-off value- is of importance in order to assess response to therapy [4, 5, 15]. That fact makes the need for accuracy of the actual CTC count more imperative.

Towards that direction, we previously reported the use of image analysis algorithms to eliminate the operator bias and automatically identify CTCs in the CellSearch generated images [16]. Efforts to improve these algorithms have continued and have led to the image analysis program ACCEPT. ACCEPT enables a quantitative definition of objects, such as CTCs and tdEVs, derived from the images using specific parameters. In the present study, an excellent correlation (R^2^ = 0.88) was found (Supplementary Figure 2) between the manual (obtained by the operator) and the automated CTC counts (obtained by ACCEPT).

To define the cut-off values dichotomizing patients into groups of higher and lower risk by ACCEPT automated CTC and tdEV counts, a retrospective data set of 84 CRPC patients from the original IMMC38 study was used. ROC analysis in that data set (Supplementary Figure 3) showed a cut-off value of 5 CTCs in 7.5 mL of whole blood that stratified patients into favorable and unfavorable groups (Figure 3). The CTC cut-off was validated in a prospective data set of 45 newly enrolled CRPC patients. In the full data set, patients of the unfavorable group (with ≥ 5 CTCs) had significantly shorter median OS compared to patients of the favorable group (*p <* 0.001) with a HR of 2.8 (95% CI: 1.8 to 4.4).

In the CellSearch image analysis algorithms, objects expressing both Cytokeratin as well as DAPI are presented to the operator, whereas the majority of tdEVs is missed, as the latter ones do not have DAPI signal. Using the automated ACCEPT identification of tdEVs however, the labor-intensive manual review of the original stack of 144–180 of fluorescence images for each patient sample [7] can be replaced by a process with perfect repeatability. In our study, there was a strong correlation between automated CTC and tdEV counts (Supplementary Figure 2) and their presence in higher amounts was strongly correlated with poor clinical outcome (Figure 3).

Since tdEVs were isolated with the CellSearch system, they were enriched from the blood fraction centrifuged at 800 *g* based on their EpCAM expression and were detected by the expression of Cytokeratins. However, the tdEV number in the plasma should be much higher because the vast majority of these EVs have a diameter below 4 µm [17]. Moreover, the CellSearch Analyzer was not designed for the detection of small particles, and the fraction of tdEVs below the CellSearch detection limit is unknown. Further investigation is recommended for the isolation and detection of tdEVs from plasma of patients and their correlation with clinical outcome.

To assess the clinical relevance of soluble Cytokeratins in plasma, CK18 and ccCK18 were determined by M65 and M30 ELISAs respectively. Several studies have shown the significantly elevated (cc)CK18 levels in serum/plasma of different cancer patients compared to the respective values of healthy donors [24–26]. Moreover, CK18 and ccCK18 have been used in several studies to evaluate the induced cell death modes and antitumor activity of different drug treatments [8, 18–21]. Interestingly, in a previous study, both ccCK18 and CK18 levels in plasma were predictive of the clinical outcome of small cell lung cancer patients [22]. In another study on both small and non-small cell lung cancer patients, the serum levels of ccCK18 were prognostic for OS [23]. A third study demonstrated significantly increased levels of ccCK18 in the sera of breast cancer patients compared to HDs but without any association of ccCK18 with the OS of these patients [26]. In our study in CRPC patients, we found that both CK18 and ccCK18 are elevated in patients compared to HDs, but only CK18 is prognostic for OS (Figure 4). It is worth mentioning that the ELISA assays we applied for CK18 and ccCK18 do not involve a detergent and thus measure only the soluble proteins and do not detect CK18 or ccCK18 inside tdEVs. Adding a detergent to the plasma may solubilize the tdEVs, and potentially result in a prognostic ccCK18 because a portion of cytokeratins in CTCs and tdEVs is caspase-cleaved as shown by M30 expression [27].

In summary, our findings suggest that ACCEPT software allows fast enumeration of well defined objects as CTCs and tdEVs eliminating interoperator bias. The enumeration of tdEVs in CRPC patients can provide prognostic information equivalent to CTCs in CRPC disease. tdEVs were typically detected at a 20 times higher frequencies in 7.5 ml of blood compared to CTCs so tdEVs may offer increased utility. The association between OS and CK18 in CRPC is not as strong as CTCs and tdEVs based on the respective HRs. The multivariable analysis of the full data set of CRPC patients including CTCs, tdEVs, CK18 and ccCK18 and traditional biomarkers such as age, PSA, ALP, LDH, Alb and Hb resulted in a final model with LDH, albumin, hemoglobin and tdEVs. tdEVs improved significantly the prognostic prediction of the patients. Neither CK18 nor ccCK18 were predictive of OS after LDH inclusion. CTCs were highly correlated with tdEVs so were not present in the final multivariable model but provided equivalent prognostic information. To predict whether a certain treatment will be effective, assessment of the treatment target will need to be assessed. Preferably one would examine tumor cells representing the various metastatic sites, but as that is practically not feasible, CTCs can provide this information provided that one can isolate them in sufficient quantity [28, 29]. Due to the fact that most patients have single-digit CTC counts, the higher number of tdEVs could render them to a promising surrogate biomarker for the assessment of changes of tumor load (through their rises and declines) in response to therapy over time and even in personalized therapeutics by proceeding with the downstream analysis of their protein and RNA cargo.

## MATERIALS AND METHODS

### Patients and healthy donors

Blood from 16 anonymous HDs was obtained after written informed consent. These samples were used to determine the baseline values of CTCs, tdEVs, CK18 and ccCK18.

All patients had histologically confirmed metastatic prostate cancer progressing despite castrate levels of testosterone and had provided written informed consent to trial protocols approved by the institutional review boards at each participating center. Two patient cohorts enrolled in different studies and during different time periods were included in the present study. More specifically, the retrospective data set consisted of 84 CRPC patients who were starting a new line of therapy and were enrolled in the IMMC-38 study [1] out of the 231 evaluable patients of IMMC38 study, because stored EDTA plasma of only these patients was available for further (cc) CK18 assessment. The digitally stored CellSearch images were also available.

The prospective data set consisted of 45 CRPC patients who were starting a new line of therapy at the Royal Marsden Hospital, and from whom stored plasma samples in CellSave and digitally stored CellSearch images were available.

### Sample collection and preparation

For the retrospective data set, 7.5 mL of blood was collected in CellSave blood collection tubes (Menarini, Huntingdon Valley, PA, USA) and 5–10 mL of blood in in EDTA tubes. For CTC and tdEV assessment, CellSave blood was processed with the CellSearch Autoprep within 96 hours from the time of blood draw. For CK18 and ccCK18 assessment, EDTA blood was centrifuged for 10 minutes at 1710 *g* without brake within 24 hours from the time of blood draw and plasma was collected and stored at –80° C until further use. The plasma samples of the retrospective data set were assessed for CK18 and ccCK18 within 5 years from their collection.

For both the prospective data set as well as the healthy donors, 7.5 mL of blood was collected in CellSave tubes, and was processed within 96 hours after collection. More specifically, the blood samples were centrifuged for 10 minutes at 800 *g* without brake and 0.5–2.0 mL of plasma was collected without disturbing the buffy coat, and stored at –80° C directly after collection until further use. The remaining blood sample was processed with the CellSearch system. The plasma samples of the HDs and the prospective data set were assessed for CK18 and ccCK18 levels within 1 year from their collection.

### Isolation and detection of CTCs and tdEVs

The CellSearch system (Menarini, Huntingdon Valley, PA, USA) was used to isolate and detect CTCs and tdEVs. The system consists of the CellTracks Autoprep^®^ and the CellTracks Analyzer II^®^. The CellTracks Autoprep^®^ immunomagnetically enriches EpCAM+ objects from blood and stains the enriched objects with the nuclear dye DAPI, phycoerythrin conjugated antibodies against cytokeratin 8, 18 and 19 (CK-PE) and allophycocyanin conjugated antibody against the leukocyte specific marker CD45 (CD45-APC). The enriched labeled objects are contained in a cartridge, which is placed in a CellTracks Magnest. The CellTracks Analyzer II^®^ captures digital images with four different fluorescent channels using a 10 ×/0.45 NA objective and a charge-coupled device camera with 6.7 × 6.7 µm sized pixels. For each cartridge, 144–180 4-layer tiff images of DAPI, FITC, CK-PE, CD45-APC are saved.

### Enumeration of CTCs and tdEVs

The CTCs and tdEVs, were enumerated using the open-source ACCEPT software (http://github.com/LeonieZ/ACCEPT) developed in the frames of CTCTrap (www.utwente.nl/en/tnw/ctctrap/) and CANCER-ID EU (www.cancer-id.eu) programs. Briefly, the digitally stored CellSearch fluorescence images are processed by ACCEPT to identify objects using multiscale segmentation [10]. Objects can be categorized using configurable criteria, and objects within a category are shown in a gallery of images as well as in scatter plots using the various parameters measured from the objects. An example of the scatterplots and images of CTCs is shown in Figure 1. The scatterplots and images of tdEVs are shown in Supplementary Figure 1. Both gates used for CTC and tdEV enumeration are mentioned in the legends of Figure 1 and Supplementary Figure 1 respectively.

### Measurement of CK18 and ccCK18 concentrations

CK18 present in epithelial cells is cleaved by caspases during apoptosis. Thus, the amount of caspase cleaved CK18 is related to apoptosis, while the total amount of CK18 is related to the sum of CK18 due to apoptosis, necrosis and present in viable cells. Two commercially available ELISA kits, namely M65 and M30-Apoptosense (VLVBio, Nacka, Sweden) were used to measure the levels of soluble ccCK18, and CK18 respectively in plasma samples of healthy donors and CRPC patients. The aforementioned ELISA assays have been already used for clinical assays [18, 19, 22, 24, 25]. The M65 assay uses two different monoclonal antibodies to recognize intact and caspase-cleaved CK18. The M30 assay uses a monoclonal antibody to recognize the neo-epitope M30, which is exposed after caspase-cleavage of CK18. Both assays have a 96-well plate format and include 7 standards of defined antigen concentrations and both a low and a high concentration quality control (QC). The assays were performed according to manufacturer’s instructions. Briefly, technical duplicates of 25 μl were added to wells coated with a mouse monoclonal capture antibody. Next, 75 μl of Horse Radish Peroxidase HRP-conjugated detection antibody solution was added. After a 2 hour incubation at room temperature (RT) with constant shaking, five sequential washing steps were performed to remove unbound antibody. Next, 20 minutes incubation in the dark with 200 μl of ≤ 2 mM 3,3’5,5’-tetramethyl-benzidine (TMB) solution resulted in color development proportional to the antigen concentration. The reaction was stopped by the addition of 50 μl of 1M sulfuric acid and the 450 nm absorbance was measured within 5–30 minutes using a microplate reader. All values were corrected for the blank (background) absorbance. After calibration with the standards of defined antigen concentrations, the average of the technical duplicates was converted to concentration in U/L.

### Statistical analysis

Statistical analysis was performed in SPSS 23.0 (SPSS Inc., Chicago, IL, USA) and Stata v15.1 (StataCorp, College Station, Texas, USA). To determine whether CTCs, tdEVs, CK18 and ccCK18 can be related to each other through a monotonic function, we performed a two-tailed Spearman’s Rho test. To assess if there were significant differences between groups (healthy donors vs CRPC patients and retrospective vs prospective data sets) in the examined continuous variables (namely CTCs, tdEVs, CK18 and ccCK18), we performed the nonparametric Mann-Whitney *U* test. To determine the cut-off values to dichotomize the retrospective data set into favorable and unfavorable groups we used Receiver Operating Characteristic (ROC) curves treating survival time as the reference value dichotomized by the median survival time. We set dichotomization cut-off values for CTCs, tdEVs, CK18, and ccCK18 on the value that led to equal sensitivity and specificity (minimum |sensitivity-specificity|). Overall Survival (OS) was defined as the elapsed time in months between blood draw and death. The patients who were lost to follow-up were censored. Median OS was determined by Kaplan–Meier survival curves, and Kaplan–Meier survival curves were compared using the log-rank test. Cox regression models, with each dataset included as a shared frailty parameter, were used to determine univariable and multivariable hazards ratios (HR) for OS with 95% confidence intervals (CI) for each dichotomized variable.

A final multivariable Cox model with each dataset included as a shared frailty parameter was fit including all characteristics as continuous variables. CK18 and ccCK18 were included with an interaction term for each dataset to account for the different methods in plasma collection. CTC, tdEV, ALP and LDH were log transformed to achieve better model fit. Due to correlation between variables the final model was selected using forward stepwise elimination (*p*_in_= 0.05 and *p*_out_= 0.10). The value of tdEV in the model was assessed by calculating Uno *et al*. C-Index [30]. Bootstrapping (*n =* 1000) was used to calculate the 95% confidence interval and the difference, delta, between c-indeces of each of the models.

## SUPPLEMENTARY MATERIALS FIGURES AND TABLES







## Abbreviations

ALP: ALkaline Phosphatase
APC: AlloPhycoCyanin
ccCK18: caspase-cleaved CytoKeratin 18
CI: Confidence Interval
CK: CytoKeratin
CK18: CytoKeratin 18
CD45: Cluster of Differentiation 45
CRPC: Castration-Resistant Prostate Cancer
CTC: Circulating Tumor Cell
DAPI: 4’,6-DiAmidino-2-PhenylIndole
DNA: DeoxyriboNucleic Acid
ECOG ps: Eastern Cooperative Oncology Group performance status
ECTM: Experimental Centre for Technical Medicine
EDTA: EthyleneDiamineTetraacetic Acid
ELISA: Enzyme-Linked ImmunoSorbent Assay
EpCAM: Epithelial Cell Adhesion Molecule
FITC: Fluorescein IsoThioCyanate
HD: Healthy Donor
HR: Hazard Ratio
HRP: Horse Radish Peroxidase
IQR: InterQuartile Range
LDH: Lactate DeHydrogenase
OS: Overall Survival
PE: PhycoErythrin
PSA: Prostate Specific Antigen
QC: Quality Control
ROC: Receiver Operating Characteristic
RT: Room Temperature
tdEV: tumor-derived Extracellular Vesicle
TMB: 3,3’5,5’-TetraMethyl-Benzidine
