# Supplementary material for: Circulating tumor cells, tumor-derived extracellular vesicles and plasma cytokeratins in castration-resistant prostate cancer patients
Source: Oncotarget. 2018 Apr 10;9(27):19283–93. doi: 10.18632/oncotarget.25019 (PMC5922396; doi:10.18632/oncotarget.25019)
Supplement: Supplementary file 1 [file oncotarget-09-19283-s001.pdf]

Circulating tumor cells, tumor-derived extracellular vesicles and plasma cytokeratins in castration-resistant prostate cancer patients

SUPPLEMENTARY MATERIALS

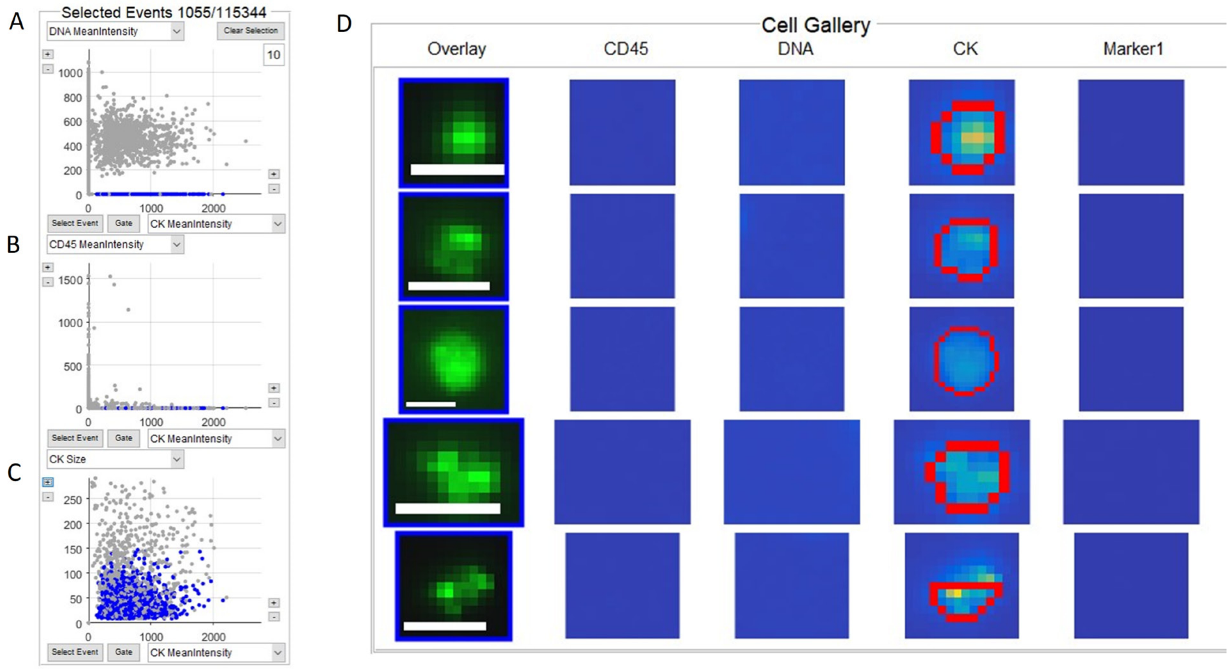

Supplementary Figure 1: ACCEPT display of tdEVs identified in a CRPC patient. Same display as used in Figure 1 with the difference that the gate for tdEV definition (Mean Intensity CD45 ≤ 5, Mean Intensity DNA ≤ 5, Mean Intensity Marker 1 ≤ 5, Mean Intensity Marker 2 ≤ 5, Mean Intensity CK > 60, Max Intensity CK > 90, Size CK ≤150, Perimeter CK > 5, Eccentricity CK ≤ 0.8, Perimeter to Area CK ≤ 1) was used on this CRPC patient. The total number of 115,344 objects is the same but the number of objects within the gate is now 1,055 as shown on top of Panel A. Panel D shows the thumbnail images of five objects that fall within the tdEV gate. Scale bar indicates 6.4 μm.

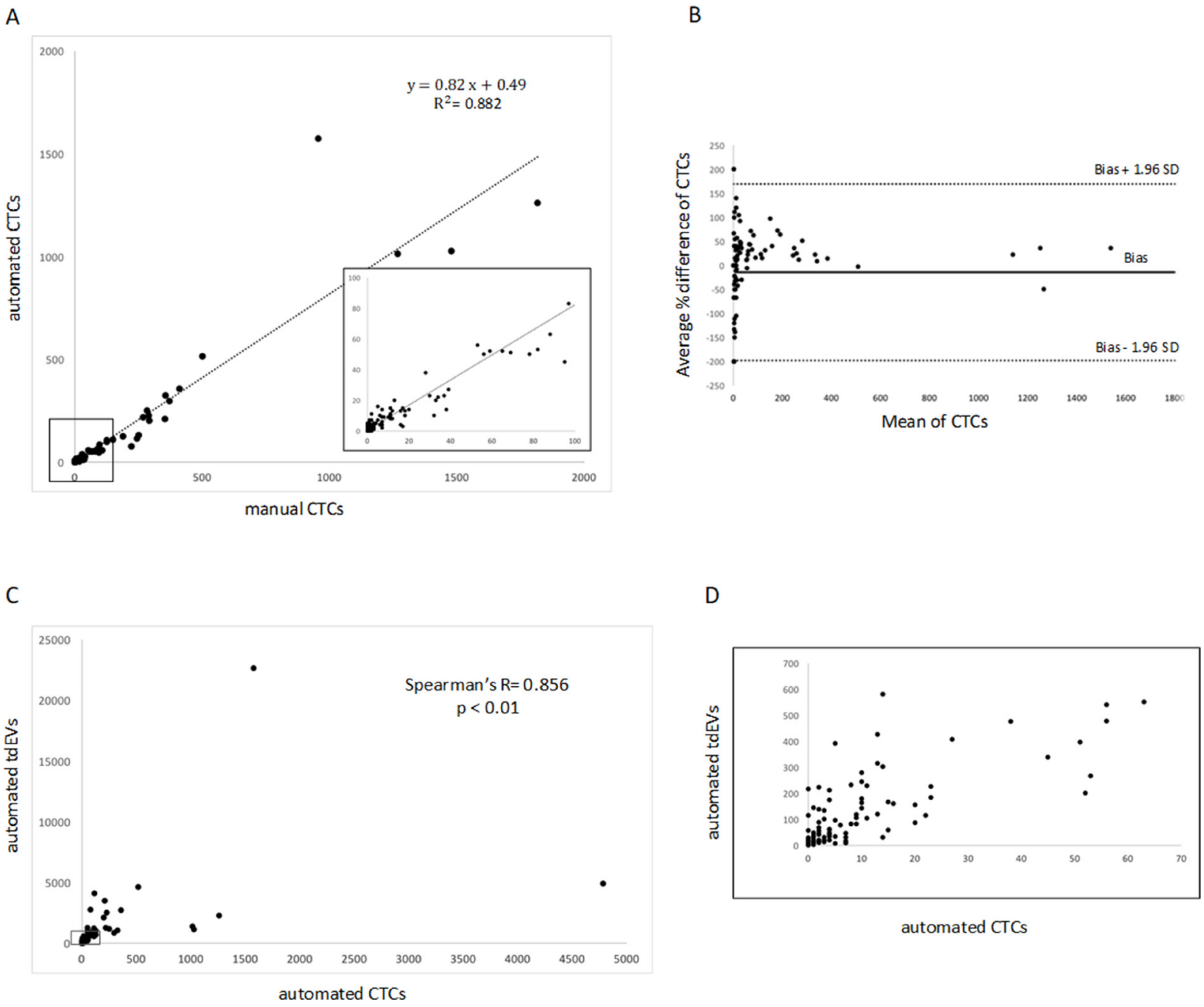

Supplementary Figure 2: Correlation of manual with automated CTCs and automated CTCs with automated tdEVs using scatterplot and Bland-Altman plot. Scatterplot (panel **A**) and Bland-Altman Plot (panel **B**) of manual CTCs (detected by operator definition) and automated CTCs (by ACCEPT enumeration). The scatterplot of the enclosed by square data points of panel A is shown in higher magnification. Scatterplot (panel **C**) of automated CTCs and automated tdEVs of 129 CRPC patients. The number of CTCs is significantly correlated with the number of tdEVs (Spearman's rho test R = 0.856, $p < 0.01$). The scatterplot of the enclosed by square data points is shown in panel (**D**).

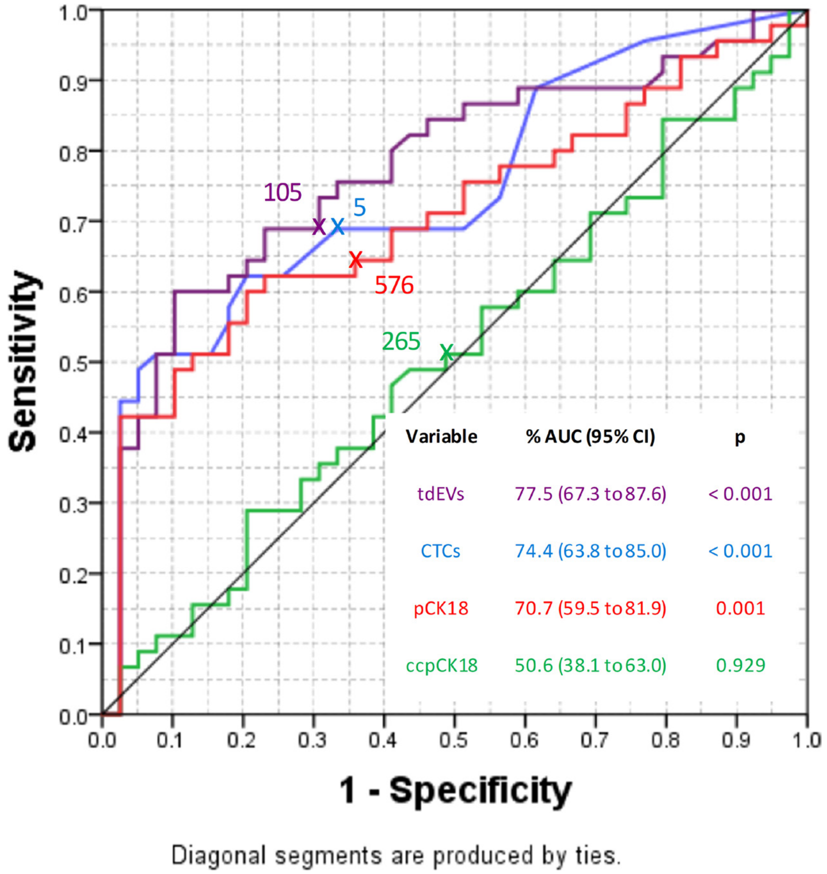

Supplementary Figure 3: ROC curves to determine the dichotomization cut-off values for CTCs, tdEVs, CK18 and ccCK18 in the retrospective data set. The x and the respective number indicates the value where the sensitivity is equal to the specificity for each biomarker (value of biomarker for which |sensitivity-specificity| is minimum).

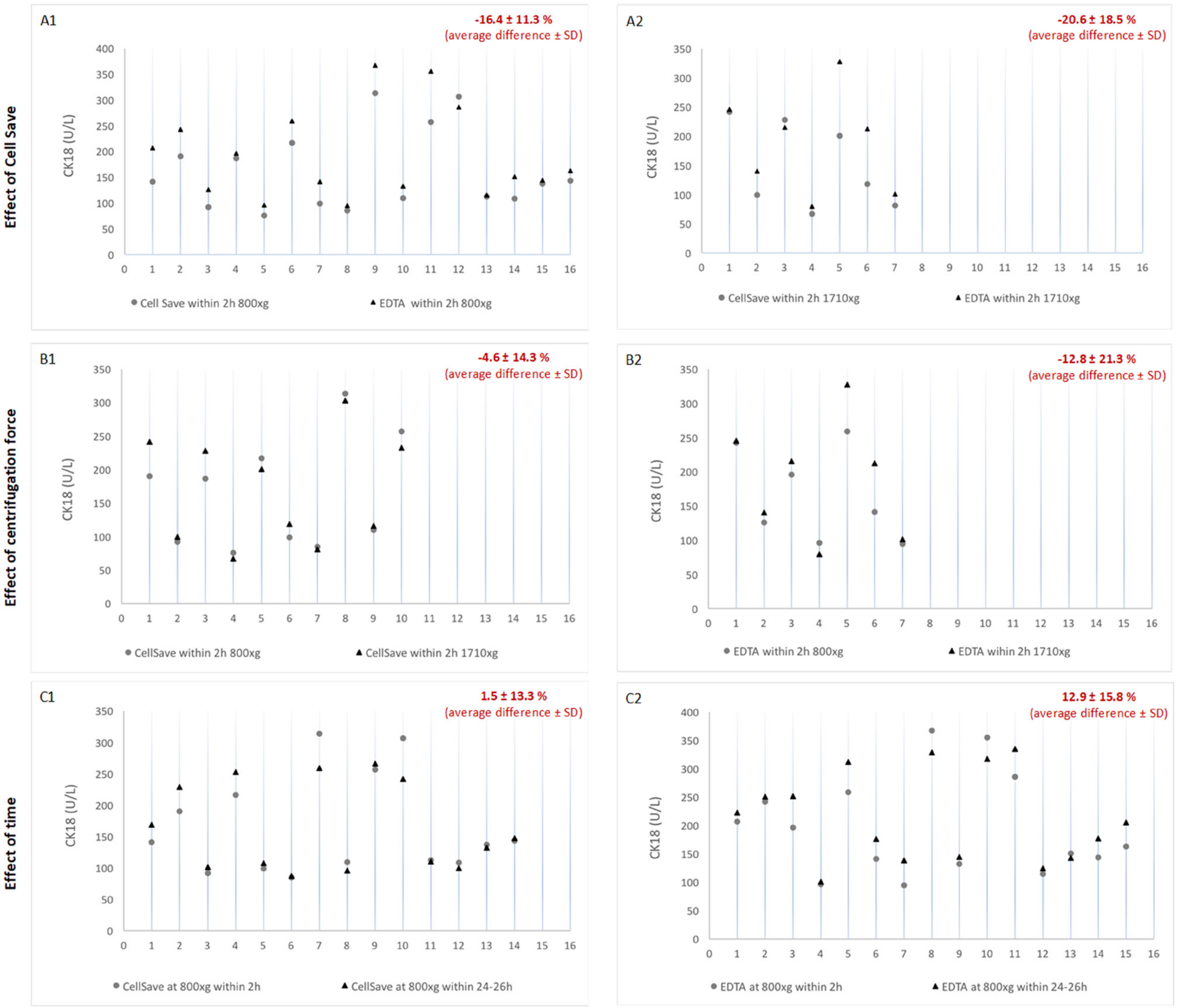

Supplementary Figure 4: The concentration of plasma CK18 measured by M65 and ccCK18 measured by M30 was significantly lower in the prospective data set compared to the retrospective data set. Parameters that could explain this difference were the blood collection tube EDTA versus CellSave (Panels **A1** and **A2**), the applied centrifugation force of 800xg versus 1710 × g (Panels **B1** and **B2**) for the plasma collection and the time between blood draw and plasma collection (Panels **C1** and **C2**). Blood samples of 16 healthy donors were collected in both EDTA and CellSave tubes and the three different parameters were tested on the resulting measured CK18. The largest influence was contributed to the blood collection tube.

Supplementary Table 1: Values of prognostic serum markers (PSA, LDH, ALP, Alb, Hb), age, Gleason score and ECOG performance status of included patients in both retro- and pro- spective data sets. Descriptive statistics (median, mean, SD, min and max) are included for each data set. See Supplementary_Table_1

Supplementary Table 2: Site of metastases and prior treatments of patients of each data set. Percentages of patients with bone, visceral metastases and prior radiation, surgery and chemotherapy are included at the end of each data set. See Supplementary_Table_2

Supplementary Table 3: Correlation between CTCs, tdEVs, CK18, ccCK18, PSA, LDH, ALP, Hb and Alb of retrospective and prospective data sets using the Spearman's Rho test

			tdEVs	CK18	ccCK18	PSA	LDH	ALP	Hb	Alb
CTCs	Correlation coefficient (2-tailed significance)	Retro-spective data set	0.821** (<0.001)	0.353* (0.001)	0.171 (0.119)	0.493** (<0.001)	0.344* (0.001)	0.503** (<0.001)	-0.433** (<0.001)	-0.154 (0.176)
		Pro-spective data set	0.932** (<0.001)	0.301 (0.044)	0.111 (0.467)	0.14 (0.378)	0.530** (<0.001)	0.656** (<0.001)	-0.171 (0.261)	-0.28 (0.062)
tdEVs	Correlation coefficient (2-tailed significance)	Retro-spective data set		0.450** (<0.001)	0.134 (0.223)	0.483** (<0.001)	0.480** (<0.001)	0.487** (<0.001)	-0.429** (<0.001)	-0.347** (<0.001)
		Pro-spective data set		0.357 (0.016)	0.195 (0.200)	0.244 (0.119)	0.589** (<0.001)	0.695** (<0.001)	-0.213 (0.161)	-0.279 (0.063)
CK18	Correlation coefficient (2-tailed significance)	Retro-spective data set			0.532** (<0.001)	0.303* (0.005)	0.357* (0.001)	0.380** (<0.001)	-0.285* (0.009)	-0.385** (<0.001)
		Pro-spective data set			0.640** (<0.001)	0.078 (0.621)	0.608** (<0.001)	0.353 (0.017)	-0.464* (0.001)	-0.257 (0.088)
ccCK18	Correlation coefficient (2-tailed significance)	Retro-spective data set				0.045 (0.681)	0.09 (0.417)	-0.058 (0.601)	0.044 (0.692)	-0.168 (0.138)
		Pro-spective data set				0.3 (0.053)	0.449* (0.002)	0.174 (0.253)	-0.098 (0.523)	-0.079 (0.606)
PSA	Correlation coefficient (2-tailed significance)	Retro-spective data set					0.300* (0.006)	0.391** (<0.001)	-0.308* (0.005)	-0.224 (0.047)
		Pro-spective data set					0.074 (0.64)	0.15 (0.343)	0.08 (0.613)	-0.002 (0.988)
LDH	Correlation coefficient (2-tailed significance)	Retro-spective data set						0.308* (0.005)	-0.376* (0.001)	-0.255 (0.024)
		Pro-spective data set						0.466** (<0.001)	-0.167 (0.272)	-0.131 (0.39)
ALP	Correlation coefficient (2-tailed significance)	Retro-spective data set							-0.263 (0.017)	-0.380* (0.001)
		Pro-spective data set							-0.186 (0.221)	-0.174 (0.252)
Hb	Correlation coefficient (2-tailed significance)	Retro-spective data set								0.314* (0.005)
		Pro-spective data set								0.433* (0.003)

**correlation is significant at 0.001 level, *correlation is significant at 0.01 level.

Supplementary Table 4: Univariable Cox proportional Hazards regression analysis of age, PSA, ALP, LDH, CK18, ccCK18, tdEVs and CTCs for retrospective, prospective and full data sets (Enter method)

variable	Data set	Cut-off	HR	95% CI for HR		*p*
				lower	upper	
tdEVs	retrospective	105	3.6	2.1	6.3	< 0.001
	prospective	105	5.0	2.2	11.1	< 0.001
	full	105	3.8	2.4	5.9	< 0.001
CTCs	retrospective	5	2.6	1.5	4.3	< 0.001
	prospective	5	4.0	1.8	9.1	< 0.001
	full	5	2.8	1.8	4.4	< 0.001
Hemoglobin (Hb)	retrospective	12.55	0.3	0.2	0.6	< 0.001
	prospective	12.55	0.8	0.3	2.0	0.566
	full	12.55	0.4	0.2	0.6	< 0.001
CK18	retrospective	576	2.4	1.4	4.0	0.001
	prospective	232	3.5	1.6	7.8	0.001
	full	both	2.5	1.6	3.8	< 0.001
Prostate specific antigen (PSA)	retrospective	87	2.3	1.4	3.8	0.002
	prospective	87	1.7	0.8	3.7	0.192
	full	87	2.0	1.3	3.0	0.002
Alkaline phosphatase (ALP)	retrospective	126	2.2	1.3	3.6	0.004
	prospective	126	2.7	1.2	6.0	0.017
	full	126	2.1	1.4	3.3	0.001
Albumin (Alb)	retrospective	3.95	0.5	0.3	0.9	0.009
	prospective	3.95	0.8	0.3	2.122	0.658
	full	3.95	0.6	0.3	0.9	0.010
Lactate dehydrogenase (LDH)	retrospective	230	1.9	1.1	3.1	0.015
	prospective	230	2.7	1.2	5.8	0.014
	full	230	2.0	1.3	3.0	0.002
Age (per 10 years)	retrospective		1.2	0.9	1.6	0.407
	prospective		1.0	0.6	1.6	0.889
	full		1.1	0.9	1.4	0.484
ccCK18	retrospective	265	1.2	0.7	2.0	0.483
	prospective	81	1.3	0.6	2.8	0.484
	full	both	1.2	0.8	1.8	0.460
