# Supplementary material for: Circulating tumor cells, tumor-derived extracellular vesicles and plasma cytokeratins in castration-resistant prostate cancer patients
Source: Oncotarget. 2018 Apr 10;9(27):19283–93. doi: 10.18632/oncotarget.25019 (PMC5922396; doi:10.18632/oncotarget.25019)
Supplement: Supplementary file 2 [file oncotarget-09-19283-s002.docx]

**Supplementary Table 1:** Values of prognostic serum markers (PSA, LDH, ALP, Alb, Hb), age, Gleason score and ECOG performance status of included patients in both retro- and pro- spective data sets. Descriptive statistics (median, mean, SD, min and max) are included for each data set.

| **Retrospective data set** | | | | | | | | |
| --- | --- | --- | --- | --- | --- | --- | --- | --- |
| **Patient #** | **Age** | **Gleason score** | **ECOG ps** | **PSA (ng/mL)** | **LDH (U/mL)** | **ALP (U/mL)** | **Alb (g/dL)** | **Hb (g/dL)** |
| R1 | 67 | 9 | 0 | 62 | 146 | 228 |  | 14.4 |
| R2 | 56 | 7 | 0 | 17.7 | 175 | 90 |  | 12.8 |
| R3 | 69 | 6 | 0 | 3796 | 232 | 82 | 4.4 | 10.4 |
| R4 | 62 | 7 |  | 62.9 | 467 | 179 | 4 | 10.6 |
| R5 | 60 | 9 | 1 | 29.7 | 215 | 78 |  | 12.4 |
| R6 | 58 | 7 | 2 | 1443 | 361 | 409 | 4 | 9.7 |
| R7 | 79 |  | 2 | 320 | 939 | 252 | 3.4 | 8.6 |
| R8 | 74 | 9 | 1 | 175 | 384 | 917 | 3.9 | 12.4 |
| R9 | 65 | 8 | 2 | 527 | 324 | 230 | 3.6 | 8.2 |
| R10 | 77 | 8 | 1 | 247 | 243 | 103 | 3.7 | 11.6 |
| R11 | 70 | 8 | 1 | 122 | 285 | 99 | 4.4 | 11.8 |
| R12 | 82 | 5 | 1 | 1634 | 237 | 130 | 3.9 | 9.5 |
| R13 | 66 | 8 | 0 | 33.3 | 248 | 96 | 4.4 | 13.7 |
| R14 | 81 | 8 | 1 | 129 | 198 | 89 | 4 | 12.7 |
| R15 | 72 | 7 | 2 | 88 | 614 | 83 | 4.4 | 11.9 |
| R16 | 72 | 9 | 1 | 80 | 214 | 129 | 4 | 11.5 |
| R17 | 61 | 8 | 1 | 64 | 225 | 145 | 4.3 | 13.6 |
| R18 | 70 | 7 | 0 | 219 | 411 | 387 | 3.5 | 9.8 |
| R19 | 70 | 7 | 0 | 756 | 224 | 104 | 3.6 | 11.3 |
| R20 | 76 | 7 | 0 | 60 | 220 | 97 | 3.4 | 11.5 |
| R21 | 58 | 7 | 1 | 18.5 | 182 | 126 | 3.8 | 12.3 |
| R22 | 59 | 8 | 0 | 29.8 | 644 | 68 | 3.3 | 11.9 |
| R23 | 58 | 7 | 0 | 76 | 258 | 125 | 3.8 | 13.7 |
| R24 | 78 | 4 | 0 | 268 | 272 | 94 | 3.5 | 15 |
| R25 | 74 | 9 | 1 | 135 | 1117 | 696 | 3.9 | 11.6 |
| R26 | 64 | 9 | 0 | 73 | 193 | 102 | 3.9 | 13.6 |
| R27 | 64 | 7 | 0 | 64 | 298 | 135 | 4.5 | 13.4 |
| R28 | 63 | 9 | 0 | 24.7 | 229 | 63 | 4.1 | 13 |
| R29 | 87 | 7 | 1 | 241 | 305 | 219 | 4.2 | 11.1 |
| R30 | 81 | 8 | 0 | 61 | 188 | 450 | 3.8 | 11.1 |
| R31 | 70 | 7 | 1 | 965 | 659 | 321 | 4 | 9.2 |
| R32 | 76 | 9 | 1 | 47 | 115 | 127 | 4.1 | 13.2 |
| R33 | 72 | 7 | 0 | 481 | 235 | 337 | 3.9 | 14 |
| R34 | 53 | 6 | 2 | 107 | 348 | 136 | 3.2 | 13.2 |
| R35 | 67 | 7 | 0 | 39.3 | 258 | 223 | 3.4 | 13.4 |
| R36 | 77 | 9 | 1 | 36 | 157 | 144 | 4 | 13.2 |
| R37 | 86 | 2 | 1 | 26.5 | 200 | 67 | 3.5 | 12 |
| R38 | 75 | 7 | 0 | 18.2 | 177 | 92 | 4.2 | 13.2 |
| R39 | 82 | 6 | 1 | 49 | 264 | 170 | 3.5 | 12 |
| R40 | 85 | 8 | 2 | 127 | 660 | 180 | 3.5 | 10.3 |
| R41 | 65 | 10 | 0 | 580 | 533 | 51 | 3.9 | 11.5 |
| R42 | 79 | 8 | 1 | 82 | 270 | 169 | 3.8 | 13.7 |
| R43 | 81 | 6 | 0 | 90 |  |  | 3.5 | 11.1 |
| R44 | 63 | 7 | 0 | 28.7 | 159 | 47 | 3.8 | 13 |
| R45 | 81 | 5 | 1 | 285 | 114 | 205 | 4.1 | 12.1 |
| R46 | 72 | 2 | 0 | 107 | 158 | 65 | 4.1 | 12.7 |
| R47 | 75 | 9 | 0 | 786 | 188 | 86 | 3.5 | 11.7 |
| R48 | 56 | 9 | 0 | 36.1 | 169 | 81 | 3.6 | 11 |
| R49 | 68 | 9 | 1 | 114 | 206 | 186 | 3.7 | 12 |
| R50 | 74 | 8 | 1 | 178 | 222 | 99 | 4.3 | 14.3 |
| R51 | 79 | 7 | 1 | 1118 | 272 | 165 |  | 12.3 |
| R52 | 87 | 7 | 1 | 3630 | 311 | 227 | 3.6 | 13.6 |
| R53 | 63 | 9 | 0 | 37.8 | 247 | 385 | 3.6 | 12.2 |
| R54 | 68 | 8 | 1 | 227 | 182 | 585 |  | 9.5 |
| R55 | 85 | 7 | 1 | 604 | 1040 | 352 | 2.8 | 8.7 |
| R56 | 80 | 7 | 0 | 17.4 | 208 | 79 | 3.8 | 11.3 |
| R57 | 76 | 9 | 1 | 69 | 146 | 87 | 3.3 | 13.5 |
| R58 | 70 |  | 0 | 58 | 181 | 170 | 4 | 11 |
| R59 | 71 | 7 | 0 | 23.6 | 237 | 124 | 4.4 |  |
| R60 | 63 | 8 | 0 | 334 | 206 | 67 | 4.5 | 12.7 |
| R61 | 81 | 8 | 0 | 11.3 | 234 | 85 | 4.7 | 15.6 |
| R62 | 80 | 8 | 0 | 491 | 204 | 63 | 4.5 | 14.8 |
| R63 | 77 | 6 | 1 | 86 | 205 | 85 | 4.3 | 13.1 |
| R64 | 68 | 6 | 1 | 49 | 255 | 120 | 3.9 | 13.1 |
| R65 | 60 | 7 | 0 | 210 | 168 | 182 | 3.7 | 13.6 |
| R66 | 86 |  | 0 | 10.5 | 260 | 118 | 4.4 | 14.7 |
| R67 | 75 |  | 0 | 186 | 138 | 250 | 3.9 | 14 |
| R68 | 75 | 8 | 0 | 19.2 | 284 | 164 | 4.2 | 9.4 |
| R69 | 69 |  | 0 | 435 | 263 | 103 | 3.8 | 13 |
| R70 | 69 | 6 | 0 | 90 | 219 | 46 | 4.2 | 13.4 |
| R71 | 60 | 9 | 1 | 29.2 | 347 | 45 | 4.5 | 11.3 |
| R72 | 58 | 8 | 1 | 5842 | 436 | 495 | 3.5 | 12.1 |
| R73 | 61 | 6 | 0 | 107 | 212 | 106 | 4.4 | 13.4 |
| R74 | 60 | 9 | 0 | 10.1 | 203 | 103 | 4.4 | 13.1 |
| R75 | 65 | 8 | 1 | 551 | 222 | 287 | 3.7 | 9.5 |
| R76 | 49 | 8 | 1 | 27.3 | 210 | 118 | 3.7 | 11.9 |
| R77 | 65 | 7 | 1 | 23.9 | 185 | 82 | 39 | 13.5 |
| R78 | 58 | 7 | 0 | 73 | 167 | 144 | 4.2 | 14.2 |
| R79 | 57 | 6 | 1 | 512 | 173 | 310 | 4 | 13.54 |
| R80 | 75 |  |  | 475 | 244 | 240 | 3.2 | 11.5 |
| R81 | 62 | 8 | 0 | 29.8 | 157 | 78 | 4.8 | 14.8 |
| R82 | 56 | 7 | 1 | 683 | 2092 | 1801 | 3.1 | 10.7 |
| R83 | 61 | 9 | 2 | 1162 | 234 | 181 | 4.3 | 11.4 |
| R84 | 64 | 9 | 1 | 20.9 | 310 | 223 | 3.5 | 13.6 |
| **Mean** | 69.8 | 7.4 | 0.6 | 384.4 | 308.3 | 198.3 | 4.3 | 12.2 |
| **SD** | 9.1 | 1.5 | 0.6 | 868.7 | 271.5 | 231.5 | 4 | 1.6 |
| **Median** | 70 | 7.5 | 1 | 89 | 232 | 127 | 3.9 | 12.3 |
| **Min** | 49 | 2 | 0 | 10.1 | 114 | 45 | 2.8 | 8.2 |
| **Max** | 87 | 10 | 2 | 5842 | 2092 | 1801 | 39 | 15.6 |
|  | | | | | | | | |
| **Prospective data set** | | | | | | | | |
| **Patient #** | **Age** | **Gleason score** | **ECOG ps** | **PSA (ng/mL)** | **LDH (U/mL)** | **ALP (U/mL)** | **Alb (g/dL)** | **Hb (g/dL)** |
| P1 | 75 | 6 | 1 | 16 | 256 | 82 | 3.3 | 13 |
| P2 | 68 | 7 | 1 | 220 | 356 | 93 | 3.9 | 10.9 |
| P3 | 75 | 6 | 1 | 28 | 140 | 56 | 3.4 | 12.5 |
| P4 | 83 | 8 | 1 | 280 | 146 | 75 | 3.1 | 11.1 |
| P5 | 60 | 9 | 2 | 56 | 199 | 243 | 3.3 | 10.2 |
| P6 | 69 | 8 | 1 | 623 | 160 | 93 | 3.5 | 10.4 |
| P7 | 62 | 7 | 0 | 111 | 202 | 72 | 3.8 | 12.1 |
| P8 | 71 | 9 | 1 | 23.4 | 207 | 93 | 3.9 | 12.4 |
| P9 | 60 | 7 | 1 | 204 | 553 | 213 | 3.3 | 10.2 |
| P10 | 70 | 7 | 1 | 11 | 156 | 64 | 3.3 | 11 |
| P11 | 61 | 7 | 2 | 154 | 314 | 2130 | 3.3 | 10.7 |
| P12 | 55 | 8 | 1 | 26 | 322 | 787 | 2.9 | 10.9 |
| P13 | 71 | 9 | 2 | 152 | 209 | 115 | 3.1 | 10 |
| P14 | 75 | 7 | 1 | 26 | 150 | 77 | 3.7 | 11.5 |
| P15 | 76 | 6 | 1 | 5.9 | 514 | 103 | 3.3 | 11 |
| P16 | 74 | 7 | 0 | 100 | 237 | 46 | 4.1 | 13.2 |
| P17 | 68 | 9 | 1 | 6 | 155 | 64 | 3.6 | 9.5 |
| P18 | 71 | 10 | 1 | 101 | 343 | 99 | 3.5 | 12.1 |
| P19 | 72 | 9 | 1 | 15.4 | 215 | 162 | 2.9 | 10.1 |
| P20 | 65 | 9 | 0 | 120 | 185 | 162 | 4.1 | 13.6 |
| P21 | 75 | 7 | 0 | 17 | 219 | 75 | 4.1 | 15.8 |
| P22 | 62 | 9 | 0 | 129 | 174 | 47 | 4.4 | 13.9 |
| P23 | 73 | 10 | 1 | 70 | 176 | 123 | 3.2 | 9.6 |
| P24 | 77 | 7 | 1 | 34 | 203 | 186 | 3.8 | 12.6 |
| P25 | 63 | 6 | 1 | 73 | 148 | 52 | 3.7 | 8.6 |
| P26 | 79 |  | 2 | 180 | 1786 | 1282 | 2.3 | 9 |
| P27 | 67 | 9 | 1 | 70 | 391 | 146 | 3.7 | 11.1 |
| P28 | 68 |  | 1 | 2510 | 140 | 166 | 3.3 | 12.7 |
| P29 | 76 | 9 | 1 |  | 429 | 717 | 3.2 | 10.4 |
| P30 | 53 |  | 1 | 8.8 | 201 | 73 | 3.6 | 9.9 |
| P31 | 71 | 9 | 1 | 95 | 371 | 67 | 3.2 | 12.1 |
| P32 | 75 |  | 1 |  | 234 | 236 | 3.6 | 11.5 |
| P33 | 56 | 9 | 2 | 161 | 313 | 61 | 3.2 | 11.3 |
| P34 | 69 | 9 | 2 | 130 | 508 | 1425 | 4 | 9.4 |
| P35 | 62 |  | 1 | 32 | 257 | 305 | 4.3 | 12.3 |
| P36 | 71 | 7 | 1 | 162 | 520 | 88 | 3.2 | 11.2 |
| P37 | 56 | 7 | 0 | 86 | 173 | 59 | 3.3 | 11.4 |
| P38 | 68 | 9 | 1 | 115 | 197 | 104 | 3.4 | 12.5 |
| P39 | 71 | 10 | 1 | 5 | 188 | 79 | 3.3 | 8.6 |
| P40 | 65 |  | 1 |  | 568 | 4027 | 3.7 | 11.5 |
| P41 | 64 | 7 | 1 | 22 | 268 | 82 | 3.7 | 12.3 |
| P42 | 61 | 9 |  | 750 | 170 | 54 | 3.9 | 12.5 |
| P43 | 67 | 9 | 1 | 338 | 395 | 81 | 3.5 | 11.5 |
| P44 | 54 | 8 | 0 | 214 | 169 | 272 | 3.8 | 13.7 |
| P45 | 49 |  | 1 | 133 | 238 | 86 | 4 | 8.1 |
| **Mean** | 67.4 | 8 | 1 | 181.3 | 299 | 327.2 | 3.5 | 11.3 |
| **SD** | 7.6 | 1.2 | 0.5 | 397.6 | 257.3 | 697.3 | 0.4 | 1.6 |
| **Median** | 68 | 8 | 1 | 97.5 | 215 | 93 | 3.5 | 11.3 |
| **Min** | 49 | 6 | 0 | 5 | 140 | 46 | 2.3 | 8.1 |
| **Max** | 83 | 10 | 2 | 2510 | 1786 | 4027 | 4.4 | 15.8 |

*SD: Standard Deviation, min: minimum, max: maximum, PSA: Prostate Specific Antigen, LDH: Lactate DeHydrogenase, ALP: ALkaline Phosphatase, Hb: Hemoglobin, Alb: Albumin, ECOG ps: Eastern Cooperative Oncology Group performance status*
