# Supplementary material for: Circulating tumor cells, tumor-derived extracellular vesicles and plasma cytokeratins in castration-resistant prostate cancer patients
Source: Oncotarget. 2018 Apr 10;9(27):19283–93. doi: 10.18632/oncotarget.25019 (PMC5922396; doi:10.18632/oncotarget.25019)
Supplement: Supplementary file 3 [file oncotarget-09-19283-s003.docx]

**Supplementary Table 2:** Site of metastases and prior treatments of patients of each data set. Percentages of patients with bone, visceral metastases and prior radiation, surgery and chemotherapy are included at the end of each data set

| **Retrospective data set** | | | | | | |
| --- | --- | --- | --- | --- | --- | --- |
| **Patient #** | **Site of metastases** | **Prior radiation** | **Prior surgery** | **Prior chemotherapy** | **Abiraterone** | **Enzalutamide** |
| R1 | bone | no | yes | no | no | no |
| R2 | bone and visceral | yes | yes | no | no | no |
| R3 | bone and visceral | yes | no | yes | no | no |
| R4 | bone and visceral | yes | yes | no | no | no |
| R5 | visceral | yes | yes | no | no | no |
| R6 | bone and visceral | yes | yes | no | no | no |
| R7 | bone and visceral | yes | no | yes | no | no |
| R8 | bone | yes | yes | yes | no | no |
| R9 | bone and visceral | yes | yes | yes | no | no |
| R10 | bone | no | yes | yes | no | no |
| R11 | visceral | yes | yes | no | no | no |
| R12 | bone | yes | yes | no | no | no |
| R13 | bone | yes | yes | no | no | no |
| R14 | bone and visceral | yes | no | no | no | no |
| R15 | bone and visceral | yes | yes | no | no | no |
| R16 | bone | no | no | yes | no | no |
| R17 | bone | yes | yes | yes | no | no |
| R18 | bone and visceral | yes | yes | yes | no | no |
| R19 | bone | no | no | yes | no | no |
| R20 | bone | yes | yes | yes | no | no |
| R21 | bone and visceral | yes | yes | no | no | no |
| R22 | bone and visceral | yes | yes | yes | no | no |
| R23 | bone | yes | yes | yes | no | no |
| R24 | bone and visceral | yes | yes | yes | no | no |
| R25 | bone | yes | yes | yes | no | no |
| R26 | bone and visceral | no | yes | yes | no | no |
| R27 | bone | no | yes | no | no | no |
| R28 | no | no | no | no | no | no |
| R29 | bone | no | yes | no | no | no |
| R30 | bone | no | no | no | no | no |
| R31 | bone | no | no | no | no | no |
| R32 | bone | yes | yes | no | no | no |
| R33 | bone | no | yes | no | no | no |
| R34 | bone | yes | yes | yes | no | no |
| R35 | bone and visceral | yes | no | yes | no | no |
| R36 | bone | no | no | no | no | no |
| R37 | no | yes | yes | no | no | no |
| R38 | bone | no | yes | no | no | no |
| R39 | bone | yes | yes | yes | no | no |
| R40 | bone and visceral | yes | yes | yes | no | no |
| R41 | bone | no | no | yes | no | no |
| R42 | ?/visceral | yes | no | no | no | no |
| R43 | bone | no | yes | no | no | no |
| R44 | bone | no | yes | yes | no | no |
| R45 | bone | yes | yes | no | no | no |
| R46 | bone | yes | yes | yes | no | no |
| R47 | bone and visceral | yes | no | no | no | no |
| R48 | visceral | yes | yes | no | no | no |
| R49 | bone | no | no | no | no | no |
| R50 | bone | yes | no | yes | no | no |
| R51 | bone | no | yes | no | no | no |
| R52 | bone | no | yes | no | no | no |
| R53 | bone | yes | yes | no | no | no |
| R54 | bone | yes | yes | no | no | no |
| R55 | bone | yes | no | no | no | no |
| R56 | bone | no | yes | no | no | no |
| R57 | bone and visceral | no | yes | yes | no | no |
| R58 | bone | no | yes | yes | no | no |
| R59 | bone | yes | no | no | no | no |
| R60 | bone | yes | yes | yes | no | no |
| R61 | no | no | no | no | no | no |
| R62 | bone | yes | yes | no | no | no |
| R63 | bone | yes | yes | no | no | no |
| R64 | bone | yes | no | no | no | no |
| R65 | bone and visceral | no | no | no | no | no |
| R66 | bone | no | yes | no | no | no |
| R67 | bone | no | yes | no | no | no |
| R68 | bone | yes | yes | no | no | no |
| R69 | bone and visceral | no | yes | no | no | no |
| R70 | bone | yes | yes | no | no | no |
| R71 | visceral | yes | yes | yes | no | no |
| R72 | bone and visceral | no | no | no | no | no |
| R73 | bone | no | yes | no | no | no |
| R74 | bone and visceral | no | no | yes | no | no |
| R75 | bone and visceral | yes | no | no | no | no |
| R76 | bone and visceral | yes | no | yes | no | no |
| R77 | ?/no visceral | yes | yes | no | no | no |
| R78 | bone | yes | yes | no | no | no |
| R79 | bone | no | yes | no | no | no |
| R80 | no | yes | yes | no | no | no |
| R81 | bone and visceral | no | yes | no | no | no |
| R82 | bone | no | yes | no | no | no |
| R83 | bone | yes | no | yes | no | no |
| R84 | bone | yes | yes | no | no | no |
| **Summary** | 88.1% bone metastases | 60.7% yes | 70.2% yes | 35.70% | 0% yes | 0% yes |
|  | 34.5% visceral metastases |  |  |  |  |  |
| **Prospective data set** | | | | | | |
| **Patient #** | **Site of metastases** | **Prior radiation** | **Prior surgery** | **Prior chemotherapy** | **Abiraterone** | **Enzalutamide** |
| P1 | bone | yes | no | yes | yes | yes |
| P2 | bone | no | no | yes | yes | yes |
| P3 | bone and visceral | yes | no | yes | yes | yes |
| P4 | bone | no | no | yes | yes | no |
| P5 | bone | no | no | yes | yes | no |
| P6 | bone | yes | no | yes | no | yes |
| P7 | no | yes | no | no | yes | no |
| P8 | bone | yes | no | yes | yes | no |
| P9 | bone and visceral | no | no | yes | yes | no |
| P10 | bone | yes | no | yes | yes | no |
| P11 | bone | no | no | yes | yes | no |
| P12 | bone | yes | no | yes | yes | no |
| P13 | bone | yes | no | yes | yes | no |
| P14 | bone | yes | no | yes | yes | no |
| P15 | bone | yes | no | yes | yes | yes |
| P16 | bone | yes | no | no | yes | no |
| P17 | bone | yes | no | yes | yes | no |
| P18 | bone | yes | no | yes | no | yes |
| P19 | bone | no | no | yes | yes | no |
| P20 | bone | no | no | yes | yes | yes |
| P21 | bone | yes | no | yes | yes | no |
| P22 | no | no | no | yes | yes | no |
| P23 | bone | no | no | yes | yes | no |
| P24 | bone | no | no | yes | yes | yes |
| P25 | bone | yes | no | yes | yes | no |
| P26 | bone and visceral | no | no | yes | yes | no |
| P27 | bone and visceral | yes | no | yes | no | yes |
| P28 | bone | no | no | yes | yes | yes |
| P29 | bone and visceral | no | no | yes | yes | yes |
| P30 | bone and visceral | no | no | yes | no | yes |
| P31 | bone | no | no | yes | yes | yes |
| P32 | bone | yes | yes | yes | no | yes |
| P33 | visceral | no | no | yes | yes | no |
| P34 | bone | no | yes | yes | yes | no |
| P35 | bone | no | no | yes | yes | yes |
| P36 | bone | yes | no | yes | yes | yes |
| P37 | visceral | yes | no | yes | no | yes |
| P38 | bone and visceral | no | no | yes | yes | no |
| P39 | bone and visceral | yes | no | yes | yes | yes |
| P40 | bone | no | yes | ? | ? | ? |
| P41 | visceral | yes | no | no | no | yes |
| P42 | bone | yes | no | yes | yes | yes |
| P43 | bone | yes | no | yes | yes | no |
| P44 | bone | yes | no | yes | yes | no |
| P45 | bone | no | no | yes | yes | yes |
| **Summary** | 88.9% bone metastases | 53.3% yes | 6.7% yes | 91.1% yes | 82.2% yes | 46.7% yes |
|  | 24.4% visceral metastases |  |  |  |  |  |
